# Association between the Level of Physical Activity and Health-Related Quality of Life in Type 1 Diabetes Mellitus. A Preliminary Study

**DOI:** 10.3390/jcm10245829

**Published:** 2021-12-13

**Authors:** Adrián Domínguez-Domínguez, Ismael Martínez-Guardado, Francisco Javier Domínguez-Muñoz, Sabina Barrios-Fernandez, Jesús Morenas-Martín, Miguel Angel Garcia-Gordillo, Jorge Carlos-Vivas

**Affiliations:** 1 Promoting a Healthy Society Research Group (PHeSO), Faculty of Sport Sciences, University of Extremadura, 10003 Cáceres, Spain; adomingugu@alumnos.unex.es (A.D.-D.); jorgecv@unex.es (J.C.-V.); 2 Faculty of Life and Nature Sciences, Nebrija University, 28015 Madrid, Spain; 3 Physical Activity and Quality of Life Research Group (AFYCAV), Faculty of Sport Sciences, University of Extremadura, 10003 Cáceres, Spain; 4 Social Impact and Innovation in Health (InHEALTH) Research Group, Faculty of Sport Sciences, University of Extremadura, 10003 Cáceres, Spain; sabinabarrios@unex.es; 5 Motor Control Research Group, Faculty of Sport Sciences, University of Extremadura, 10003 Cáceres, Spain; jesusmorenas@unex.es; 6 Universidad Autónoma de Chile, Sede Talca 3467987, Chile; miguelgarciagordillo@gmail.com

**Keywords:** diabetes mellitus, physical activity, fitness, health-related quality of life

## Abstract

Type 1 Diabetes Mellitus (T1D) is a chronic autoimmune disease characterized by the selective destruction of the beta cells of the pancreas causing an absolute deficiency of insulin for life. This pathology carries associated risks so that it is essential to measure Health-Related Quality of Life (HRQoL) in this population. The aim was to analyse associations between the level of physical activity and HRQoL in people with T1D. The sample consisted of 172 participants with T1D diagnoses, between 18 and 49 years (31.29 ± 8.17). The participants answered different questionnaires related to physical activity (IPAQ) and HRQoL (EsDQOL, ViDa1, 15D, and EQ-5D-5L). The results showed significant correlations between the level of physical activity and HRQoL. Vigorous physical activity had an impact on the HRQoL questionnaires, such as the well-being dimension (rho = 0.349; *p* < 0.001) of the ViDa1 questionnaire. A significant correlation between total physical activity and levels of anxiety and depression was observed: anxiety (15D) (rho = 0.328; *p* < 0.001) and anxiety/depression (EQ-5D-5L) (rho = 0.324; *p* < 0.001). The present study showed associations between higher levels of physical activity and higher HRQoL parameters in the population with T1D, which can be erected as a reason for exercise prescription in these patients.

## 1. Introduction

Diabetes Mellitus (DM) affects millions of people around the world and currently has an increasing prevalence driven by a complex interplay of socioeconomic, demographic, environmental and genetic factors. The high social impact of DM, characterized by premature mortality and low quality of life due to its complications, has a significant global economic impact for both countries and health systems, as well as for people with DM and their families [1]. Among the different types of DM, Type 2 Diabetes Mellitus (T2D) is the most common, representing around 90% of cases worldwide; 5–10% have Type 1 Diabetes Mellitus (T1D) and the rest, Gestational Diabetes Mellitus (GDM) or specific types of diabetes (MODY, neonatal, etc.) [2]. 

T1D is an autoimmune disease caused by the selective immune destruction of the beta cells of the pancreas located in the islets of Langerhans, causing an absolute deficiency of insulin for life [3]. Thus, the diagnosis of T1D occurs when: fasting blood glucose is ≥126 mg/dL, blood glucose is ≥200 mg/dL and there are symptoms such as polyphagia, polydipsia, polyuria, and/or the result of Glycosylated Haemoglobin (HbA1c) is ≥6.5%.

Physical exercise constitutes a suitable strategy to reduce costs and increase health parameters in people with T1D [4]. Regular physical activity practice is associated with benefits in cardiovascular and lipid profiles, glycaemic control (decrease in daily insulin dose and HbA1c), and fitness [5]. Thus, regular exercise reduces LDL cholesterol and triglycerides and increases HDL cholesterol, which can reduce the risk of heart attacks and strokes [6]. Concerning the benefits and recommendations of physical activity for the different stages of life, participation in sports activities during the first years of life has a considerable effect on glycaemic variables during adulthood [7]. Physical exercise is associated with a better Health-Related Quality-of-Life (HRQoL), and the lack of physical exercise in adulthood causes greater use of medical resources, which is why it is recommended to promote exercise in this population [8]. Previous studies have also analysed the role of metabolism in HRQoL [9,10,11], so promoting healthy and sporting habits from an early age and adolescence is necessary [12,13]. However, during the transition to adulthood, young adults with T1D struggle to maintain a balance between the demands of managing the disease and their lives, so it can be very beneficial to regularly assess HRQoL [14].

Health-Related Quality of Life (HRQoL) is an important indicator that focuses on the individuals’ subjective self-perception about their current health status and ability to perform activities in different life domains, considering individuals’ subjective insights of their well-being, physical, social and mental health, and functioning [15,16]. As it is an important indicator of health status, HRQoL should be studied extensively. Numerous studies have been done on T2D, with much fewer studies researching the T1D population [17,18]. People with T1D have a worse HRQoL as their age increases [19], with the management of severe hypoglycaemia and the prevention of complications being crucial [20]. Therefore, this study aims to explore existing correlations between the level of physical activity and HRQoL in a population with T1D diagnosis.

## 2. Materials and Methods

### 2.1. Study Design

A cross-sectional design was used; participants underwent a single online evaluation, completing a series of questionnaires to analyse different parameters related to HRQoL.

### 2.2. Participants

Before the participant recruitment, a sample size estimation was conducted. A sample size of 104 achieves 90% power to detect a difference of −0.3 between the null hypothesis correlation of 0.09 (insignificant correlation) [21] and the alternative hypothesis correlation of 0.39 (weak correlation) [21] using a two-sided hypothesis test with a significance level of 0.05.

The following eligibility criteria were considered: (a) being diagnosed with T1D and (b) being 18–49 years old. Although initially 228 people completed the questionnaires, 56 were excluded because they did not meet the eligibility criteria. Finally, the sample was composed of 172 participants (Table 1). 

### 2.3. Instruments

The International Physical Activity Questionnaire (IPAQ): aims to provide a common and international tool to compare physical activity practice data. Its short version was used, which provides information on the time spent walking, in activities of moderate and vigorous intensity, and sedentary activities [22]. This short version has been tested extensively, including studies with T1D population [23]. One MET (Metabolic Equivalent Task) is equal to energy expenditure during rest and is approximately equal to 3.5 mL O_2_ kg^−1^ min^−1^ in adults [24]. In this study, moderate intensity was defined as 600 MET/week, or 3.57 MET-h/week and vigorous intensity was defined as ≥1500 MET/week or 8.92 MET-h/week. 

The Spanish Version of the Diabetes Quality of Life (EsDQOL) [25]: although the original version had 46 items, the Spanish version consisted of 43 items divided into four dimensions: satisfaction (15 questions), impact (17 questions), social/vocational concern (7 questions), and concern related to DM (4 questions). Responses were quantified using a five-point Likert scale. It has excellent reliability for T1D population (Cronbach’s alpha of 0.90) [26].

The T1D Quality of Life (ViDa1): is a specific instrument to measure HRQoL in people with T1D [27]. The ViDa1 has 34 items grouped into four different dimensions: interference in life (12 items), self-care (11 items), well-being (6 items), and concern about the disease (5 items). The response options are given through five levels. The ViDa1 can allow for obtaining a total score per subscale. Their four dimensions have demonstrated good internal consistency, with Cronbach’s alpha values between 0.71 and 0.86 [27].

The EuroQol-5 Dimensions-5 Levels (EQ-5D-5L): is a generic instrument to measure self-perceived health, which can be used both in healthy individuals and people with pathologies. This tool consists of a Visual Analogue Scale (VAS) from 0 to 100, and a questionnaire with five dimensions (mobility, self-care, performing of usual activities, pain/discomfort, and anxiety/depression) with five response levels [28]. Studies support the EQ-5D-5L descriptive system and the EQ-5D-5L index validity for T1D population [29].

The 15D questionnaire [30]: is a generic, comprehensive, and self-administered questionnaire composed of 15 dimensions: mobility, vision, hearing, breathing, sleeping, eating, speech, excretion, usual activities, mental function, discomfort and symptoms, depression, distress, vitality, and sexual activity. Each dimension corresponds to an item with five response levels with 1 being the best, and 5 being the worst. The questionnaire’s total score, which represents the health status, is reached by the sum of all dimensions.

### 2.4. Procedures

Before starting the study, all participants were informed about the study procedures. They all provided signed informed consent following the ethical standards of the Declaration of Helsinki. The procedure was approved (92/2021) by the Institutional Ethics Committee of The University of Extremadura (Spain).

The questionnaires were transcribed into an electronic form to be distributed by email. The form was open for 30 days to respond. Once this period was over, the electronic form was closed and data were obtained for further analysis.

### 2.5. Statistical Analysis

SPSS 25 for Windows (SPSS Inc., Chicago, IL, USA) was used to conduct the statistical analyses. The Kolmogorov–Smirnov test was used to check the distribution of data not following a normal distribution. The Spearman correlation coefficient was used to establish correlations between the results of the IPAQ and the different HRQOL questionnaires. Schober’s thresholds classification was followed to interpret the correlation coefficient: 0.10 to 0.39, weak; 0.4 to 0.69, moderate; 0.70 to 0.89, strong; and ≥0.9, very strong [21]. 

## 3. Results

Table 2 shows the correlation between physical activity and the result of the different dimensions of the EsDQOL questionnaire. A weak significant correlation was observed between METs of vigorous physical activity and the total score of the EsDQOL.

Table 3 shows the association between all the dimensions and the total score of the ViDa1 questionnaire and physical activity. The well-being dimension correlates weakly and positively with most physical activity parameters.

Overall, results show how both questionnaires are weakly related to HRQoL in people with T1D: EsDQOL (Table 2) and ViDa1 (Table 3). There is a clear impact regarding vigorous activity, as most of their dimensions weakly correlate with vigorous physical activity, except to “interference with life” and “concern for disease” dimensions.

Table 4 shows the associations between the total score and dimensions of the 15D questionnaire and physical activity practice. There are weak isolated correlations between the 15D dimensions and some variables of physical activity. Thus, the results indicate that depression, anxiety, and vitality are the ones most affected by the level of physical activity. 

Table 5 presents the correlation between physical activity and the different dimensions of EQ-5D-5L as well as their total score and the EQ-5D-5L VAS on current health status.

## 4. Discussion

The main contribution of this study is the finding of significant relationships between physical activity and different HRQoL dimensions in a population with T1D. These results are supported by previous studies, which also found associations between these variables in populations with T1D of different life stages: children [31], adolescents [32], and older adults [8].

Vigorous physical activity showed the highest associations on the different dimensions and outcomes of the HRQoL questionnaires. Moreover, in the EsDQOL and the ViDa1 questionnaires, specifics for the T1D population, associations were found in satisfaction, well-being, and self-care. These results seem logical, as the practice of physical activity is associated with numerous benefits in terms of T1D care [6]. Furthermore, intense physical activity has the greatest association, therefore it should be considered as included in the physical activity prescription for the T1D population [12]. Regarding total physical activity, our data indicates a high association impact on the different aspects related to HRQoL, including anxiety and depression (15D and EQ-5D-5L questionnaires). Therefore, the practice of physical activity seems to be an important factor for the prevention and management of anxiety and depression in T1D population [31]. Liu et al. [33] explored the relationship between anxiety, depression, and HRQoL in using the EQ-5D-5L questionnaire, finding similar results on the anxiety/depression dimension (42%). 

Finally, regarding the correlations between physical activity and other HRQoL dimensions, there were some variables with weak association (e.g., sex) or they did not even exist (e.g., speech or eating). This is probably since most of the participants do not have any problems in certain HRQoL dimensions, as is the case, for example, with the eating or the speech dimension of the 15D questionnaire. 

Therefore, the practical implication of the present study supports that the prescription of physical activity in the population with T1D could be an adequate strategy to enhance HRQoL and disease care. However, it needs to be confirmed through a longitudinal study that analyses the effect of physical activity practice on HRQoL in the T1D population. Therefore, the causal relationship between physical activity level and HRQoL cannot be clarified in this study because it follows a cross-sectional design.

Nevertheless, this study presents several limitations: the wide difference that exists between the participants’ sex (77% women vs. 23% men); information was not collected about participants’ previous treatment; the study has been performed without carrying out comparations with T2D or a healthy population, so no cause-effect relationships could be obtained, and only online questionnaires were applied, so the sample could be formed by respondents with biases [34]. Likewise, the cross-sectional nature of the manuscript does not obtain data such as if participants were used to exercising, how long and at which level they had been exercising before, and consider performing a multivariate analysis. Thus, it would be interesting for future studies to include laboratory tests (such as the assessment of pancreatic islets β cells activity by measuring c-peptide) that could correlate with the results of the questionnaires. It would also be interesting for future research to perform subgroup analysis by sex or disease duration that was not possible here due to the subgroup analysis, which could not be carried out since at least one of the fragmentation subgroups did not reach a value sufficient to achieve a power of 80% or more, despite exceeding the previously estimated sample size.

## 5. Conclusions

This study emphasizes the importance of physical activity in terms of better HRQoL of people with T1D, since participants who perform more physical activity had greater self-perception of both their health and their care of the disease. 

Vigorous physical activity would be related to greater self-perception of T1D care, so the prescription of this type of exercise may provide benefits in terms of the perception of the disease. It has also been observed that higher total physical activity has a positive impact on anxiety and depression levels.

## Figures and Tables

**Table 1:** Participant characteristics.

Characteristics	Total = 172		Women = 132		Men = 40	
	Mean	SD	Mean	SD	Mean	SD
Age (years)	31.29	8.17	30.05	7.64	35.38	8.64
Body weight (kg)	68.84	14.08	65.53	12.63	79.75	13.22
Height (cm)	166.46	8.96	163.21	6.78	177.18	6.65
BMI (kg/m^2^)	24.74	4.08	24.56	4.28	25.33	3.31
HbA1c (%)	6.68	0.73	6.71	0.74	6.60	0.68
T1D years (years)	14.51	9.51	14.36	9.30	15.00	10.27
Current Health Status (VAS)	74.0	14.1	73.0	14.5	76.8	12.5

BMI: body mass index; HbA1c: glycosylated haemoglobin; T1D: Type 1 Diabetes Mellitus; VAS: visual analogue scale (0, “the worst possible health status” to 100, “the best possible best health status”); SD: standard deviation.

**Table 2:** Correlations between EsDQOL and IPAQ questionnaires in Type 1 Diabetes Mellitus population (*n* = 172).

	Vigorous Activity (METs)		Moderate Activity (METs)		Walking (METs)		Total Activity (METs)		Days of Physical Activity (Number)		Total Activity (min/sem)	
	Rho	*p*	Rho	*p*	Rho	*p*	Rho	*p*	Rho	*p*	Rho	*p*
EsDQOL (total score)	−0.257 **	0.001	−0.179 *	0.019	−0.065	0.395	−0.217 **	0.004	−0.194 *	0.011	−0.203 **	0.008
Satisfaction	−0.271 **	0.000	−0.144	0.059	−0.097	0.206	−0.220 **	0.004	−0.126	0.099	−0.252 **	0.001
Impact	−0.133	0.082	−0.067	0.384	−0.033	0.670	−0.100	0.194	−0.039	0.608	−0.129	0.093
Concern Social/vocational	−0.198 **	0.009	−0.103	0.177	−0.001	0.987	−0.130	0.089	−0.165 *	0.030	−0.091	0.237
Diabetes-related concern	−0.157 *	0.040	−0.149	0.052	−0.129	0.092	−0.221 **	0.004	−0.216 **	0.005	−0.178 *	0.019

** Correlation is significant at 0.01 level (bilateral); * correlation is significant at 0.05 level (bilateral). ESDQOL: Spanish version of diabetes quality of life questionnaire; METs: metabolic equivalent task; Rho: Spearman correlation coefficient. IPAQ: The International Physical Activity Questionnaire.

**Table 3:** Correlations between ViDa1 and IPAQ questionnaires in the Type 1 Diabetes Mellitus population (*n* = 172).

	Vigorous Activity (METs)		Moderate Activity (METs)		Walking (METs)		Total Activity (METs)		Days of Physical Activity (Number)		Total Activity (min/sem)	
	Rho	*p*	Rho	*p*	Rho	*p*	Rho	*p*	Rho	*p*	Rho	*p*
ViDa1 (total score)	0.262 **	0.001	0.200 **	0.008	0.123	0.107	0.239 **	0.002	0.161 *	0.035	0.239 **	0.002
Interference with life	−0.091	0.233	−0.156 *	0.040	−0.037	0.632	−0.064	0.404	0.018	0.814	−0.153 *	0.045
Self-care	0.215 **	0.005	0.102	0.181	0.211 **	0.005	0.239 **	0.002	0.090	0.239	0.216 **	0.004
Welfare	0.349 **	0.000	0.296 **	0.000	0.084	0.273	0.305 **	0.000	0.246 **	0.001	0.286 **	0.000
Concern for disease	−0.106	0.167	−0.027	0.730	−0.008	0.916	−0.073	0.341	−0.118	0.124	−0.072	0.349

** Correlation is significant at 0.01 level (bilateral); * correlation is significant at 0.05 level (bilateral). ViDa1: health-related quality of life questionnaire for Type-1 Diabetes; METs: metabolic equivalent task; Rho: Spearman correlation coefficient.

**Table 4:** Correlations between 15D and IPAQ questionnaires in the Type 1 Diabetes Mellitus population (*n* = 172).

	Vigorous Activity (METs)		Moderate Activity (METs)		Walking (METs)		Total Activity (METs)		Days of Physical Activity (Number)		Total Activity (min/sem)	
	Rho	*p*	Rho	*p*	Rho	*p*	Rho	*p*	Rho	*p*	Rho	*p*
15D (total score)	−0.215 **	0.005	−0.217 **	0.004	−0.094	0.221	−0.196 *	0.010	−0.126	0.099	−0.288 **	<0.001
Mobility	0.047	0.542	−0.016	0.834	−0.173 *	0.024	−0.034	0.656	−0.115	0.134	−0.031	0.688
Vision	−0.044	0.571	−0.088	0.250	−0.245 **	0.001	−0.187 *	0.014	−0.185 *	0.015	−0.227 **	0.003
Hearing	−0.135	0.077	−0.114	0.138	−0.115	0.135	−0.126	0.098	−0.153 *	0.044	−0.142	0.063
Breathing	−0.166 *	0.030	−0.115	0.134	−0.036	0.644	−0.114	0.135	−0.117	0.127	−0.128	0.095
Sleep	−0.112	0.144	−0.112	0.145	−0.020	0.799	−0.101	0.188	−0.086	0.261	−0.149	0.052
Eating	N/A	N/A	N/A	N/A	N/A	N/A	N/A	N/A	N/A	N/A	N/A	N/A
Speech	0.023	0.767	−0.038	0.621	−0.075	0.326	0.046	0.551	0.080	0.297	−0.014	0.852
Elimination	−0.121	0.113	−0.109	0.154	−0.023	0.765	−0.087	0.255	−0.073	0.343	−0.039	0.610
Habitual activities	−0.085	0.267	−0.002	0.982	−0.136	0.075	−0.127	0.098	−0.166 *	0.029	−0.105	0.172
Mental function	−0.035	0.645	−0.166 *	0.030	−0.041	0.590	−0.082	0.284	−0.062	0.417	−0.116	0.131
Discomfort and symptoms	−0.002	0.984	0.017	0.823	−0.093	0.225	−0.051	0.507	0.051	0.508	−0.126	0.102
Depression	−0.264 **	<0.001	−0.213 **	0.005	−0.058	0.452	−0.202 **	0.008	−0.106	0.166	−0.287 **	<0.001
Anxiety	−0.170 *	0.025	−0.188 *	0.014	−0.100	0.192	−0.174 *	0.022	−0.011	0.883	−0.328 **	<0.001
Vitality	−0.194 *	0.011	−0.222 **	0.003	−0.100	0.193	−0.210 **	0.006	−0.174 *	0.023	−0.254 **	0.001
Sexual activity	−0.100	0.192	−0.092	0.230	−0.011	0.882	−0.117	0.126	−0.099	0.199	−0.158 *	0.039

** Correlation is significant at 0.01 level (bilateral); * correlation is significant at 0.05 level (bilateral); 15D: 15-D questionnaire for health-related quality of life; N/A: not applicable; METs: metabolic equivalent task; Rho: Spearman correlation coefficient.

**Table 5:** Correlations between EQ-5D-5L and IPAQ questionnaires in the Type 1 Diabetes Mellitus population (*n* = 172).

	Vigorous Activity (METs)		Moderate Activity (METs)		Walking (METs)		Total Activity (METs)		Days of Physical Activity (Number)		Total Activity (min/sem)	
	Rho	*p*	Rho	*p*	Rho	*p*	Rho	*p*	Rho	*p*	Rho	*p*
EQ-5D-5L (total score)	0.157 *	0.040	0.223 **	0.003	0.151 *	0.049	0.189 *	0.013	0.201 **	0.008	0.244 **	0.001
Mobility	0.032	0.674	−0.066	0.391	−0.073	0.339	0.003	0.967	−0.038	0.623	0.027	0.722
Personal care	−0.044	0.567	0.002	0.980	−0.107	0.164	−0.100	0.193	−0.078	0.311	−0.003	0.972
Daily activities	−0.081	0.290	−0.066	0.390	−0.098	0.202	−0.131	0.087	−0.140	0.067	−0.113	0.139
Pain/discomfort	−0.063	0.410	−0.073	0.344	−0.120	0.118	−0.095	0.216	−0.171 *	0.025	−0.075	0.329
Anxiety/depression	−0.180 *	0.018	−0.239 **	0.002	−0.105	0.171	−0.193 *	0.011	−0.103	0.179	−0.324 **	<0.001
Health status (VAS)	0.180 *	0.019	0.133	0.083	0.173 *	0.024	0.198 **	0.010	0.201 **	0.009	0.235 **	0.002

** Correlation is significant at 0.01 level (bilateral); * correlation is significant at 0.05 level (bilateral). EQ-5D-5L: Euroqol 5-dimensions questionnaire; VAS: visual analogue scale; METs: metabolic equivalent task; Rho: Spearman correlation coefficient.

## Data Availability

The datasets used during the current study are available from the corresponding author on reasonable request.
